# Cerebral Manifestation of Miliary Tuberculosis Mimicking a Parasitic CNS Infection

**DOI:** 10.1155/crnm/9275005

**Published:** 2026-10-12

**Authors:** Daniel Martens, Fatma Merzou, Nils Schroeter, Malvina Garner, Wolfgang Reith, Sergiu Groppa, Yaroslav Winter

**Affiliations:** ^1^ Department of Neurology Saarland University Homburg Germany uni-saarland.de; ^2^ Department of Neurology Saarland University Medical Center Homburg Germany uniklinikum-saarland.de; ^3^ Department of Neuroradiology Saarland University Medical Center Homburg Germany uniklinikum-saarland.de; ^4^ Department of Neurology Philipps-University Marburg Marburg Germany uni-marburg.de

**Keywords:** CNS manifestation, mimics, neurocysticercosis, toxoplasmosis, tuberculosis

## Abstract

The neuroradiological findings of central nervous system (CNS) manifestations of miliary tuberculosis can be misinterpreted as parasitic infections due to their low prevalence in the European population. We present the case of a 30‐year‐old woman in whom CNS tuberculosis was initially misdiagnosed as a parasitic infection due to the absence of pulmonary symptoms. She immigrated to Germany from Madagascar and had lived in Europe for 14 months by the time she presented to our department with an incidental MRI finding suggestive of a manifestation of toxoplasmosis. The T1‐weighted axial brain MRI scan revealed multifocal, rim‐enhancing lesions that were suggestive of parasitic infection. Following a chest CT scan that revealed miliary tuberculosis, the diagnosis was revised to neurotuberculosis. This was confirmed by a polymerase chain reaction (PCR) that detected the *Mycobacterium tuberculosis* complex in the sputum sample. The patient achieved remission following 12 months of antituberculosis therapy. In conclusion, neurotuberculosis should be considered in the differential diagnosis of infections presenting with multifocal CNS manifestations on neuroimaging scans in European patients, particularly those with a migration background.

## 1. Introduction

Tuberculosis (TB) is caused by *Mycobacterium tuberculosis*. The hallmark symptoms are cough, fever, and weight loss, but patients with TB could show mild symptoms or be asymptomatic [1]. *Mycobacterium tuberculosis* is the leading cause of infectious‐related deaths worldwide. According to the World Health Organization, 10.8 million people developed TB, and approximately 1.25 million died from the infectious disease globally in 2023 [2]. In Europe, there is a gender difference in fatality rates, with a greater impact on the male population [3]. Approximately 1% of all TB cases are attributable to miliary TB, which is characterized by widespread lymphohematogenous dissemination of bacilli‐laden foci [4]. Recent data show that cerebral involvement in miliary TB is not uncommon [5]. However, because TB is rare in European countries such as Germany, where the incidence rate is 5.3 per 100,000 inhabitants [6], cerebral manifestations of TB can be overlooked when diagnosing patients with unclear brain lesions. The same is true of other extrapulmonary manifestations of TB, which can lead to delays in diagnosis and treatment initiation [7, 8]. Another rare manifestation of TB is vasculitis [9], which, like other vasculitides triggered by infection [10, 11], requires a thorough angiological examination, including ultrasound and, if necessary, angiography.

When considering epidemiological data from Europe and making a differential diagnosis of multinodular cerebral lesions identified on a magnetic resonance imaging (MRI) scan, parasitic diseases such as toxoplasmosis and cysticercosis are more commonly suspected than neurotuberculosis, particularly if there are no pulmonary symptoms present. The incidence of seroconversion to toxoplasmosis in Germany is estimated at 1099 per 100,000 inhabitants [12]. Neurotoxoplasmosis on MRI scans is often characterized by multifocal, ring‐enhancing lesions in the cortex and periventricular white matter. This finding has been described in T1‐weighted imaging following the administration of gadolinium [13]. In some cases, an “eccentric” or “concentric target sign” has been associated with neurotoxoplasmosis, but this remains a rare finding [14].

Lesions in the parenchyma of the central nervous system (CNS) in patients with neurocysticercosis depend on the stage of the infection. Typical locations include the white and gray matter junction in the cerebral hemispheres, as well as the brainstem and the cerebellum [15].

However, the characteristic differences between these neuroinfectious diseases are not specific and can pose a major challenge in everyday clinical practice. All of these diseases frequently exhibit similar localizations on MRI scans. Signals from the T1‐weighted and T2‐weighted sequences may change over the course of the disease. Table 1 presents a comparison of the radiological features of cerebral manifestations of TB, toxoplasmosis, and cysticercosis. Patients in the early stages of the disease may exhibit mild symptoms or be asymptomatic.

**TABLE 1:** Radiological features of cerebral TB, toxoplasmosis, and cysticercosis.

Radiological feature	CNS tuberculosis	Cerebral toxoplasmosis	Neurocysticercosis
Typical location	Gray‐white junction, posterior fossa, basal meninges	Basal ganglia, corticomedullary junction, thalami	Gray‐white junction, cortex, ventricles, basal cisterns
T1	Hypointense; caseating center variable	Hypointense; hemorrhage may be hyperintense	CSF‐like cyst (vesicular); proteinaceous lesions mildly hyperintense
T2	Caseating solid tuberculomas often T2 hypointense; liquefied lesions T2 hyperintense	Hyperintense with edema; concentric target sign	Stage‐dependent; vesicular CSF‐like, colloidal hyperintense, calcified hypointense
FLAIR	Marked edema; basal meningitis if present	Marked edema	Edema mainly colloidal stage
Enhancement	Ring or nodular enhancement; conglomerate lesions	Ring enhancement; eccentric target sign	None (vesicular stage); ring enhancement (granular stage)
DWI	Tuberculous abscess often restricted diffusion; solid tuberculomas variable	Usually no marked central restriction	Usually none
SWI/GRE	Calcification or hemorrhage uncommon; susceptibility variable	Microhemorrhage possible	Excellent for calcifications/scolex
Perfusion	Low‐to‐intermediate rCBV; lower than high‐grade tumors	Low rCBV	Low perfusion
Edema	Often marked	Marked	Minimal in vesicular, marked in colloidal
Mass effect	Variable; can be marked	Common	Usually mild
Characteristic sign	T2 hypointense caseating core; conglomerate ring lesions; basal leptomeningeal enhancement	Eccentric/concentric target sign	Scolex (“hole‐with‐dot”)

*Note:* FLAIR, fluid‐attenuated inversion recovery.

Abbreviations: DWI, diffusion‐weighted imaging; GRE, gradient‐recalled echo; SWI, susceptibility‐weighted imaging.

## 2. Case Presentation

A 30‐year‐old woman born in Madagascar had immigrated to Germany 14 months before presentation and had not traveled abroad thereafter. The patient was referred to our neurological department after office‐based radiologists discovered brain lesions suggestive of a CNS manifestation of toxoplasmosis during routine MRI scans for an asymptomatic pituitary adenoma. The control imaging of the pituitary adenoma, diagnosed 2 years previously, showed no change. However, a T1‐weighted axial brain MRI scan revealed multifocal, rim‐enhancing lesions in both hemispheres, as well as in the supratentorial and infratentorial regions. These lesions were suggestive of a parasitic infection (see Figure 1A–F). The patient was asymptomatic and showed no signs of fever or pulmonary symptoms. The neurological examination revealed no abnormalities. Based on the MRI findings, the office‐based radiologists who performed the initial scan, as well as the neuroradiologists at our university hospital, suggested a parasitic infection (e.g., neurotoxoplasmosis or neurocysticercosis) as the primary diagnosis.

**FIGURE 1:**
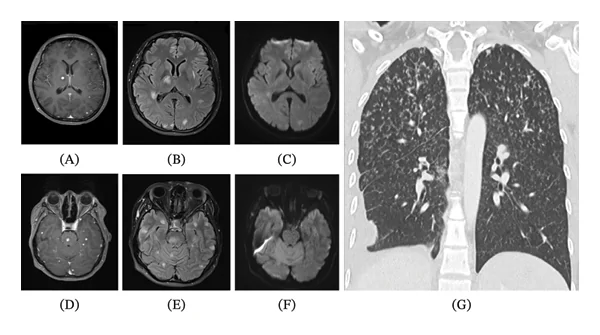
Imaging findings in a patient with neurotuberculosis. An MRI scan of the current case of neurotuberculosis reveals multiple contrast‐enhancing lesions in both hemispheres ((A) axial T1 with gadolinium) and lesions on axial FLAIR images (B) and DWI (C) images, affecting the basal ganglia and different brain lobes (frontal, parietal, and occipital). Neurotuberculosis has also affected the brainstem, as shown by axial contrast‐enhanced T1 (D), FLAIR (E), and DWI (F) images. (G) Chest CT of the current case showing bipulmonary partially confluent nodules with an apical emphasis, morphologically consistent with miliary tuberculosis.

However, a chest CT performed on the third day of hospitalization revealed multiple asymptomatic 1–3 mm bilateral pulmonary nodules (Figure 1G).

Laboratory tests revealed a normal white blood cell count but an elevated erythrocyte sedimentation rate (ESR) of 84 mm/h. The level of C‐reactive protein (CRP) was found to be 60.0 mg/L. The CD4+ T lymphocyte count was slightly reduced. Analysis of the cerebrospinal fluid (CSF) showed a normal cell count and no other notable abnormalities. The CSF culture was negative for acid‐fast bacilli. Further testing ruled out HIV and parasitic infections. Sputum cultures remained negative. However, a polymerase chain reaction (PCR) on the sputum sample detected DNA from the *Mycobacterium tuberculosis* complex. PCR of the CSF for *Mycobacterium* species was negative. A probable diagnosis of TB of the CNS was made based on radiological findings, a positive sputum PCR, and the clinical presentation (headaches, coughing, and word‐finding difficulties during the second week of hospital stay). This was further supported by the regression of cerebral lesions during the course of anti‐TB medication. Treatment was initiated according to the standard WHO RIPE (rifampicin, isoniazid, pyrazinamide, and ethambutol) regimen. Adjunctive corticosteroid therapy was initiated due to the CNS manifestation, with prednisolone administered at a dose of 60 mg per day for 4 weeks, followed by a gradual taper over the subsequent 8 weeks. Unfortunately, the patient suffered severe gastrointestinal intolerance while taking ethambutol and pyrazinamide. Instead of ethambutol and pyrazinamide, moxifloxacin and linezolid were used [16, 17]. The total duration of anti‐TB chemotherapy was set at 12 months. No paradoxical reactions occurred during the treatment or the follow‐up period. We considered the pulmonary involvement to represent the primary site of infection and the CNS lesions to constitute an extrapulmonary manifestation of TB. No other extrapulmonary manifestations of TB were identified. Laboratory controls, including monitoring of blood counts and liver and renal function, were performed every 4 weeks during antitubercular therapy. Clinical checkups to monitor for the risk of linezolid‐associated neuropathy were performed every 3 months. Following the above‐described treatment, the disease went into remission, as was observed during the 12‐month follow‐up after antitubercular therapy was discontinued. The MRI scan results were normal at this time point. The summary of this case according to CARE Case Report Guidelines is presented in Table 2.

**TABLE 2:** Summary of the clinical case according to the CARE case report guideline.

CARE item	Case summary
Patient information	30‐year‐old woman from Madagascar living in Germany for 14 months. History of asymptomatic pituitary adenoma. No recent travel, no relevant comorbidities.

Clinical findings	Incidental multifocal supra‐ and infratentorial ring‐enhancing brain lesions on routine MRI. Neurologically intact at presentation without pulmonary symptoms. Elevated ESR and CRP.

Timeline	• 2 years before admission: pituitary adenoma diagnosed. • 14 months before admission: immigration from Madagascar to Germany. • Day 0: routine MRI: multiple ring‐enhancing brain lesions; suspected neurotoxoplasmosis/neurocysticercosis. • Day 1: admission; normal neurological examination; CSF and initial microbiology unremarkable. • Day 3: chest CT demonstrated miliary pulmonary nodules. • Hospital stay: sputum PCR positive for *Mycobacterium tuberculosis* complex; diagnosis revised to probable parenchymal CNS tuberculosis. • Week 2: RIPE therapy and corticosteroids initiated. • Early treatment: ethambutol and pyrazinamide replaced by moxifloxacin and linezolid because of intolerance. • 12 months: completion of antituberculous therapy. • 12‐month follow‐up: complete clinical and radiological remission.

Diagnostic assessment	Initial differential diagnosis: cerebral toxoplasmosis and neurocysticercosis. Normal CSF including negative TB PCR. Chest CT and positive sputum PCR established probable parenchymal CNS tuberculosis associated with pulmonary miliary TB.

Therapeutic intervention	RIPE regimen plus corticosteroids. Ethambutol and pyrazinamide replaced by moxifloxacin and linezolid because of severe vomiting. Total treatment duration: 12 months.

Follow‐up and outcomes	No paradoxical reaction. Serial MRI demonstrated progressive regression of lesions. Complete clinical and radiological remission 12 months after completion of therapy.

## 3. Discussion

This case highlights the diagnostic challenges associated with CNS TB. Although TB has characteristic histopathological features, its neuroradiological manifestations are highly heterogeneous and frequently overlap with those of infectious, inflammatory, and neoplastic disorders. CNS TB may present as meningeal disease, parenchymal disease, or both. While tuberculous meningitis typically presents with meningeal irritation and abnormal CSF findings, parenchymal disease encompasses tuberculomas, cerebritis, abscesses, and encephalopathy. Miliary CNS TB usually manifests as numerous small (< 2–3 mm) noncaseating granulomas at the gray‐white matter junction that enhance homogeneously on contrast‐enhanced T1‐weighted MRI but may be inconspicuous on noncontrast scans [18].

Clinical presentation is often nonspecific, ranging from headache and seizures to focal neurological deficits or neuropsychiatric symptoms. The patient remained neurologically asymptomatic at presentation to our department, despite extensive abnormalities on MRI scans. Laboratory investigations showed only elevated ESR and CRP, suggesting an inflammatory process. The first clinical symptoms appeared during the second week of the patient’s hospital stay.

A major diagnostic challenge was the absence of CSF abnormalities. Although TB meningitis is typically associated with lymphocytic pleocytosis, elevated protein, and reduced glucose, normal CSF findings do not exclude isolated parenchymal CNS TB [19]. In cerebral tuberculomas without meningeal involvement, the inflammatory process remains confined to the brain parenchyma, and CSF examination may therefore be entirely normal. Consequently, the diagnostic yield of CSF microscopy, culture, and nucleic acid amplification tests is limited in isolated parenchymal disease [19, 20]. Even in TB meningitis, CSF smear and culture have limited sensitivity, while PCR‐based assays, although highly specific, demonstrate only moderate sensitivity. Therefore, a normal CSF examination should not delay further diagnostic evaluation when neuroimaging remains suggestive of CNS TB.

Another important diagnostic pitfall concerns the interpretation of microbiological findings obtained outside the CNS. The diagnosis in our patient was supported by a positive sputum PCR for *Mycobacterium tuberculosis*. Although a positive sputum PCR alone cannot establish the diagnosis of cerebral TB, it provides strong evidence for probable parenchymal CNS TB when combined with compatible MRI findings, the absence of a more likely alternative diagnosis, and an appropriate epidemiological background. Because brain biopsy is invasive and often impractical, the diagnosis of cerebral tuberculoma frequently relies on integrating clinical presentation, neuroimaging, extracranial microbiological evidence, epidemiological risk factors, and response to therapy.

An important aspect of this case was the stepwise differential diagnostic evaluation of multiple ring‐enhancing cerebral lesions. Based on the initial MRI findings, cerebral toxoplasmosis was considered the leading diagnosis because it represents one of the most common infectious causes of multifocal, ring‐enhancing brain lesions. *Toxoplasma gondii* infects approximately 30% of the global population [21], and seroprevalence in Germany ranges from 20% to 70% [22]. In immunocompromised patients, particularly those with HIV infection and CD4+ counts below 100 cells/mm^3^, cerebral toxoplasmosis is generally regarded as the most likely diagnosis when multiple ring‐enhancing lesions are identified [23, 24]. Moreover, cerebral toxoplasmosis and tuberculomas share several MRI characteristics, including multiple contrast‐enhancing lesions with surrounding vasogenic edema, making differentiation by conventional MRI challenging.

Neurocysticercosis was also considered because it is another important cause of multiple intracranial lesions in patients originating from endemic regions. However, several imaging features argued against this diagnosis. Typical findings of neurocysticercosis, such as cystic lesions containing an eccentric scolex, lesions at different evolutionary stages, or characteristic calcifications, were absent. Furthermore, the patient’s radiological pattern was more consistent with miliary cerebral tuberculomas than with disseminated neurocysticercosis.

Ultimately, multiple cerebral tuberculomas associated with miliary TB closely mimicked the neuroradiological appearance of cerebral toxoplasmosis, resulting in a delay in establishing the correct diagnosis. Although the patient originated from Madagascar, a country with a high TB incidence, she had been living in Germany for more than 1 year without recent travel abroad. Consequently, infectious diseases that are more prevalent in Germany were initially considered more likely, and active TB was not suspected before admission. In retrospect, greater emphasis on the patient’s epidemiological background, together with earlier microbiological investigations for *Mycobacterium tuberculosis*, might have facilitated a more timely diagnosis. The infection was not apparent prior to admission to our department. We did not use the interferon‐γ release assay (IGRA) (e.g., the T‐SPOT test) during the initial workup, which could have led to a more rapid diagnosis. Earlier use of an IGRA might have increased clinical suspicion for TB, particularly given the patient’s epidemiological background. Although IGRA cannot distinguish latent from active disease or confirm CNS involvement, a positive result could have prompted earlier targeted microbiological investigations and treatment.

Recent data show that extrapulmonary manifestations of TB are common (44%) in migrant populations from countries with a very high incidence of TB living in Europe [25]. Our patient was vaccinated with the Bacillus Calmette–Guérin (BCG) vaccine; however, this does not provide 100% protection against TB.

Despite treatment, neurotuberculosis is associated with a poor prognosis, with a mortality rate of almost 25% [26]. Fortunately, our patient achieved remission through antitubercular therapy. Severe gastrointestinal intolerance with persistent vomiting developed shortly after initiation of therapy, precluding continued administration of both pyrazinamide and ethambutol despite supportive treatment. Following consultation with infectious disease specialists, these drugs were replaced by moxifloxacin and linezolid to maintain an effective multidrug regimen while preserving adequate CNS penetration. The patient was monitored closely throughout treatment. Both moxifloxacin and linezolid achieve good CNS penetration [27, 28] and are frequently incorporated into individualized regimens when first‐line drugs cannot be used or drug toxicity necessitates modification [16, 17].

This case highlighted the importance of considering neurotuberculosis when making a differential diagnosis of infections with multifocal CNS manifestations on neuroimaging scans of European patients, particularly those with a migration background. Our intention is not to draw epidemiological conclusions from a single case but to raise awareness that cerebral TB may occur in the absence of meningeal symptoms or CSF abnormalities and closely mimic more common CNS infections such as cerebral toxoplasmosis.

## Author Contributions

Daniel Martens: investigation, resources, and writing–original draft.

Fatma Merzou and Nils Schroeter: investigation, resources, and writing–review and editing.

Malvina Garner and Wolfgang Reith: resources and writing–review and editing.

Sergiu Groppa: supervision and writing–review and editing.

Yaroslav Winter: conceptualization, investigation, resources, and writing–original draft.

## Funding

This project did not receive any specific grant from funding agencies in the public, commercial, or not‐for‐profit sectors. Open Access funding enabled and organized by Projekt DEAL.

## Ethics Statement

Ethical approval is not applicable for this clinical report as it utilized deidentified data. Written informed consent was obtained from the patient for the anonymized publication of their clinical data.

## Consent

Written informed consent was obtained from the patient for publication of this report and accompanying images.

## Conflicts of Interest

The authors declare no conflicts of interest.

## Data Availability

The data that support the findings of this study are available from the corresponding author upon reasonable request.
